# Application of nSMOL coupled with LC‐MS bioanalysis for monitoring the Fc‐fusion biopharmaceuticals Etanercept and Abatacept in human serum

**DOI:** 10.1002/prp2.422

**Published:** 2018-07-24

**Authors:** Noriko Iwamoto, Kotoko Yokoyama, Megumi Takanashi, Atsushi Yonezawa, Kazuo Matsubara, Takashi Shimada

**Affiliations:** ^1^ Leading Technology of Bioanalysis and Protein Chemistry SHIMADZU Corporation Kyoto Japan; ^2^ Department of Clinical Pharmacology and Therapeutics Kyoto University Hospital Kyoto Japan; ^3^ Graduate School of Pharmaceutical Sciences Kyoto University Kyoto Japan

**Keywords:** Abatacept, bioanalysis, clinical pharmacokinetics, Etanercept, LC‐MS, nano‐surface and molecular‐orientation limited proteolysis, therapeutic drug monitoring

## Abstract

The principle of nano‐surface and molecular‐orientation limited (nSMOL) proteolysis has a unique characteristic Fab‐selective proteolysis for antibody bioanalysis that is independent of a variety of monoclonal antibodies by the binding antibody Fc via Protein A/G in a pore with 100 nm diameter and modified trypsin immobilization on the surface of nanoparticles with 200 nm diameter. Since minimizing peptide complexity and protease contamination while maintaining antibody sequence specificity enables a rapid and broad development of optimized methods for liquid chromatography‐mass spectrometry (LC‐MS) bioanalysis, the application of regulatory LC‐MS for monitoring antibody biopharmaceuticals is expected. nSMOL is theoretically anticipated to be applicable for representative Fc‐fusion biopharmaceuticals, because Protein A/G‐binding site Fc exists on the C‐terminus, and its functional domain is available to orient and interact with the reaction solution. In this report, we describe the validated LC‐MS bioanalysis for monitoring Ethanercept and Abatacept using nSMOL technology. The quantitation range of Ethanercept in human serum was from 0.195 to 100 μg/mL using the signature peptide VFCTK (aa.43‐47), and that of Abatacept was from 0.391 to 100 μg/mL using the signature peptide MHVAQPAVVLASSR (aa.1‐14). Both proteins fulfilled the guideline criteria for low‐molecular‐weight drug compounds. The results indicate that the clinical and therapeutic monitoring for antibody and Fc‐fusion biopharmaceuticals are adequately applicable using nSMOL proteolysis coupled with LC‐MS bioanalysis.

**Abbreviation**
ADA: antidrug antibodies
AS: ankylosing spondylitis
AS: ankylosing spondylitis
CDR: complementarity‐determining region
CTLA‐4: cytotoxic T‐lymphocyte antigen 4
GMA: glycidyl methacrylate
IBD: inflammatory bowel disease
IMID: simmune‐mediated inflammatory diseases
JCA: juvenile chronic arthritis
KEGG: Kyoto Encyclopedia of Genes and Genomes
MRM: multiple reaction monitoring
nSMOL: nano‐surface and molecular‐orientation limited
PsA: psoriatic arthritis
RA: rheumatoid arthritis
TNFα: Tumor necrosis factor α IMIDs
nSMOL: nano‐surface and molecular‐orientation limited
LC‐MS: liquid chromatography‐mass spectrometry

## 1 INTRODUCTION

Tumor necrosis factor α (TNFα) is one of the proinflammatory cytokines that plays an important role in the pathogenetic signals in sepsis and several inflammatory diseases, especially playing the role of a mediator of systemic inflammation.^1^, ^2^ Recent studies focusing on TNFα signaling have indicated that endogenous TNFα is a key mediator in specific inflammatory responses.^3^, ^4^ The neutralizing TNF antagonists or monoclonal antibodies to TNF have exhibited clinically effective outcomes for many immune‐mediated inflammatory diseases (IMIDs) such as rheumatoid arthritis (RA),^5^, ^6^ inflammatory bowel disease (IBD),^7^ psoriatic arthritis (PsA),^5^ vasculitis,^8^ ankylosing spondylitis (AS),^9^ and juvenile chronic arthritis (JCA).^10^ Infliximab, Adalimumab, and Etanercept have been shown to have good therapeutic outcomes in various clinical trials since the first biopharmaceutical agents for IMID were launched in 1998.^11^ TNF blockade strategy is an extremely important option for first‐line biopharmaceuticals. Infliximab and Adalimumab have immunoglobulin G (IgG)‐based chimeric and human antibody structures, and Etanercept is a dimeric fusion protein consisting of the extracellular domain of the human p75 TNFα receptor II (TNFR) and Fc domain.^12^, ^13^

Discovered in 2005, Abatacept belongs to a new class of IMID therapeutic agents, which excludes the neutralizing proinflammatory cytokines.^14^, ^15^, ^16^, ^17^ Abatacept is a fusion protein comprising the extracellular domain of the inhibitory molecules cytotoxic T‐lymphocyte antigen 4 (CTLA‐4) and Fc of human IgG. CTLA4 has a higher affinity to CD80 and CD86 on antigen‐presenting cells than to CD28 on T cells. Abatacept selectively regulates the CD80/86 costimulatory signals for T‐cell activation, and is efficient for suppressive inflammatory observation.^18^, ^19^

All biologics and Fc‐fusion molecules are proteins, and therefore, intrinsically possess immunogenic potentials for B‐cell and T‐cell epitopes. Since therapy against IMIDs requires a long‐term and repeated administration for clinical efficacy, there is a constant potential to produce antidrug antibodies (ADA).^20^, ^21^, ^22^ The existence of ADAs in patient circulation is considered to be one of the possibilities for the decreased levels of protein pharmaceuticals in blood, the loss of efficacy, or drug‐related adverse events. Therefore, monitoring for therapeutic protein pharmaceuticals is essential for good clinical signatures.^23^ In order to adequately identify loss of clinical responses, determine dosage increase, or switch to next agents, it is important to obtain precise blood level information by therapeutic drug monitoring. Moreover, it is also known that the blood levels of drugs are influenced by the coexistence of ADAs depending on the analytical methods;^24^ hence, the advances in the universal monitoring technology are required.^25^, ^26^

**Figure 1:**
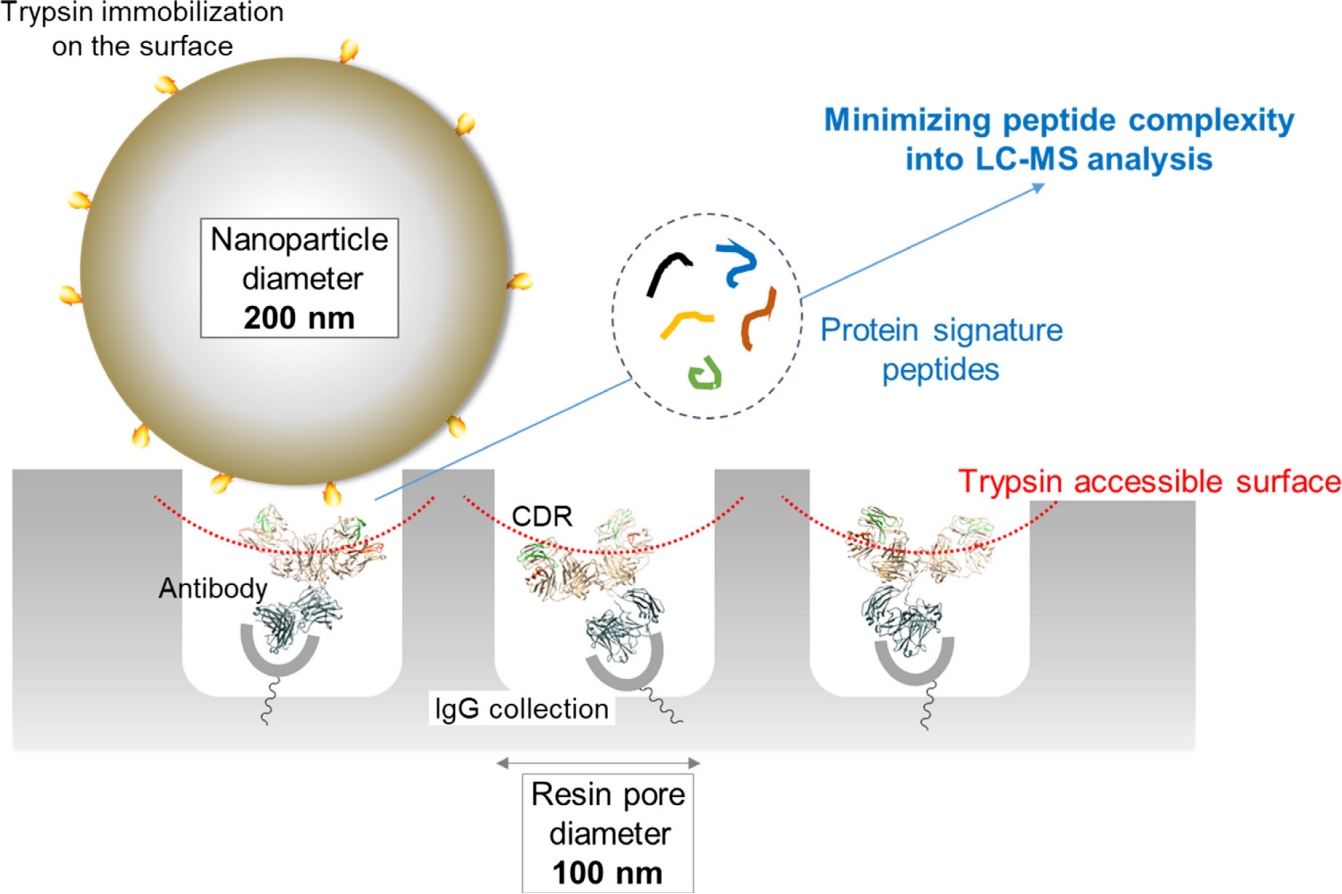
Schematic view of nSMOL reaction principle

Bioanalysis technology by LC‐MS has essential issues to overcome. LC‐MS has two main technologies such as column chromatography and mass spectrometry. In order to maintain the high‐resolution of column separation and rapid repeated analysis in LC unit, excessive sample injection should be avoided. And in MS unit, to maintain quantitative ionization, it is necessary to avoid the ionization suppression effect as much as possible and maintain an appropriate ESI interface environment. Our nano‐surface and molecular‐orientation limited (nSMOL) proteolysis principle is the sole LC‐MS bioanalysis technology (Figure 1). Briefly, protein Fc domain is first collected via Protein A/G resin with the pore diameter of 100 nm from biological samples, so that the opposite site like Fab or fused domain will be oriented to the reaction solution in the pore. And second, modified trypsin immobilized on the surface of nanoparticles with the diameter of 200 nm is reacted in this mixture of Protein A/G resin and nanoparticles. In this solid‐solid reaction field, trypsin access to the substrate is physicochemically limited. Therefore, proteolysis reaction is selectively and effectively performed on the orienting domain to the solution like the complementarity‐determining region (CDR) in IgG molecules, without a large excess of tryptic peptide matrix and extra protease contamination.^27^ The Fc‐fusion protein biopharmaceuticals described above have a human Fc domain on the C‐terminus. Therefore, nSMOL bioanalysis is theoretically applicable to Fc‐fusion protein. In this report, we describe the development of validated LC‐MS bioanalysis for monitoring Etanercept and Abatacept levels in human serum using nSMOL approach.

## 2 MATERIALS AND METHODS

### 2.1 Chemicals

Trypsin‐immobilized glycidyl methacrylate (GMA)‐coated nano‐ferrite particle FG‐beads with surface activation by N‐hydroxysuccinimide was purchased from Tamagawa Seiki (Nagano, Japan). Toyopearl AF‐rProtein A HC‐650F resin was from Tosoh (Tokyo, Japan). Etanercept was obtained from Merck Corporate (Kenilworth, NJ). Abatacept was from Bristol‐Myers Squibb (New York City, NY). Individual male and female control human serum was from Kohjin Bio (Saitama, Japan). Modified porcine trypsin and P14R (fourteen proline repeat and one arginine on C‐terminus) internal standard synthetic peptide was from Sigma‐Aldrich (St. Louis, MO). n‐octyl‐β‐D‐thioglucopyranoside (OTG) was from Dojindo Laboratories (Kumamoto, Japan). Ultrafree‐MC GV centrifugal 0.22 μm filter and ZipTip μC18 was from Merck Millipore (Billerica, MA). Other reagents, buffers, and solvents were from Sigma‐Aldrich and Wako Pure Chemical Industries (Osaka, Japan).

### 2.2 Structural confirmation of Etanercept and Abatacept peptides

After the denaturation of Etanercept or Abatacept (20 μg) in 9 mol/L urea and 2 mmol/L Tris(2‐carboxyethyl)phosphine (TCEP) at room temperature for 30 minutes, the proteins were diluted 10‐fold in 25 mmol/L Tris‐HCl buffer (pH8.0), and digested using trypsin (1 μg) at 37°C for 16 hours. The trypsin reaction was quenched by adding formic acid solution at a final concentration of 0.5%. For nSMOL reaction, 20 μg of Etanercept or Abatacept was collected with 50 μL of PBS‐substituted AF‐rProtein A resin 50% slurry in 180 μL of PBS containing 0.1% OTG with gentle vortexing at 25°C for 5 minutes. Protein A resin was collected in an Ultrafree filter device, first washed twice using 300 μL of PBS containing OTG, and then twice using 300 μL of PBS by centrifugation (10 000 *g* for 1 minute), and finally substituted with 150 μL of 25 mol/L Tris‐HCl (pH8.0) containing 0.2 mmol/L TCEP. nSMOL proteolysis was carried out using 1 μg modified trypsin on the surface of FG‐beads with gentle vortexing at 37°C for 16 hours in a saturated vapor atmosphere. After proteolysis, the reaction was stopped by adding formic acid at a final concentration of 0.5%. The peptide solution was collected by centrifugation (10 000 *g* for 1 minutes) to remove Protein A resin and trypsin FG‐beads. The structure of tryptic peptides from Etanercept and Abatacept was determined by high‐resolution liquid chromatography‐linear ion trap time‐of‐flight MS (Nexera ×2 ultra high performance liquid chromatograph and LCMS‐IT‐TOF, Shimadzu, Kyoto, Japan) and matrix‐assisted laser desorption/ionization (MALDI) TOF MS (AXIMA Performance, Shimadzu), the parent and fragment ions were assigned using an in‐house Mascot Proteome Server and Distiller peak processing software (Matrix Science, London, UK) with Etanercept (Drug Bank, http://www.drugbank.ca, accession number DB00005) and Abatacept (DB01281) amino acid sequence information. The LC‐MS conditions were as follows: solvent A, 0.1% aqueous formic acid; solvent B, acetonitrile with 0.1% formic acid; column, L‐column2 ODS, 2.1 × 150 mm, 2 μm, 10 nm pore (Chemicals Evaluation and Research Institute, Tokyo, Japan); column temperature, 40°C; flow rate, 0.2 mL/minutes; gradient program, 0‐5 minutes: %B = 3, 5‐35 minutes: %B = 3‐30 gradient, 35‐46 minutes: %B = 95, 46‐55 minutes: %B = 3. MS and MS/MS spectra were obtained using desolvation line and heat block at 250 and 400°C, respectively. Nebulizer nitrogen gas flows were set to 3 L/minutes. Drying gas pressure was 100 kPa. Ion accumulation time was 30 msec for MS, and 70 msec for MS/MS analysis. MS/MS analysis was performed using the automated data‐dependent mode. Ar pulse time into the ion trap cell was 125 μsec. The electrode of collision‐induced dissociation (CID) cell was set at −1.5 V. The MALDI MS conditions were as follows: reflectron high‐resolution mode from *m/z* 600 to 4500 mass acquisition range, high‐purity recrystallized α‐cyano‐4‐hydroxycinnamic acid as a MALDI matrix, externally calibrated by protonated mass signals of a five peptide mixture, bradykinin fragment 1‐7 (monoisotopic *m/z* 757.40), angiotensin II (*m/z* 1046.54), P14R synthetic peptide (*m/z* 1533.86), ACTH fragment 18‐39 (*m/z* 2465.20), and insulin‐oxidized B chain (*m/z* 3494.65), internally calibrated by protonated signals from tryptic autolysis fragments (*m/z* 842.51 and *m/z* 2211.10).

### 2.3 Prediction of the signature peptides of Etanercept and Abatacept

Amino acid sequences of monoclonal antibodies were obtained from Kyoto Encyclopedia of Genes and Genomes (KEGG). Multiple sequence alignment analysis was performed using the amino acid sequence of Etanercept (KEGG Drug accession D00742), Abatacept (D03203), Rituximab (D02994), and Infliximab (D02598) by ClustalW algorithm on GENETYX software (GENETYX, Tokyo, Japan). Furthermore, alignment analysis was performed on the Tumor necrosis factor receptor 2 (SwissProt accession TNR1B_HUMAN) and Cytotoxic T‐lymphocyte‐associated antigen 4 (CTLA4_HUMAN). In this analysis, theoretical tryptic peptides with no overlap to the sequence of immunoglobulin framework or original receptors and ligands, initiation of Fc‐fusion protein, positions of cysteine residue and fusion insertion were aligned.

### 2.4 Setting conditions for multiple reaction monitoring (MRM) of Etanercept and Abatacept peptides

The peptide quantitation was analyzed using an LC‐electrospray ionization‐MS (LC‐ESI‐MS) with triple quadrupole (Nexera ×2 and LCMS‐8050/8060, Shimadzu). The LC‐MS conditions were as follows: solvent A, 0.1% aqueous formic acid; solvent B, acetonitrile with 0.1% formic acid; column, Shim‐pack GISS C18, 2.1 × 50 mm, 1.9 μm, 20 nm pore (Shimadzu); column temperature, 50°C; flow rate, 0.4 mL/minute; gradient program for Etanercept, 0‐1.5 minutes: %B = 1, 1.5‐5 minutes: %B = 1‐40 gradient, 5‐6.25 minutes: %B = 95 with flow rate 1 mL/minutes, 6.25‐7 minutes: and %B = 1 with flow rate 0.4 mL/minutes; gradient for Abatacept, 0‐2 minutes: %B = 1, 2‐5 minutes: %B = 1‐35 gradient, 5‐6.4 minutes: %B = 95 with flow rate 1 mL/minutes, 6.4‐7 minutes: and %B = 1 with flow rate 0.4 mL/minutes. MS spectra were obtained with ESI probe temperature, desolvation line, and heat block at 300°C, 250°C, and 400°C, respectively. Nebulizer, heating, and drying gas flows were set to 3, 10, and 10 L/ minutes, respectively. The dwell time was set to 10 msec for each transition. MRM monitor ions of peptide fragments were imported from the measured values of structure‐assigned fragments by high‐resolution LC‐MS analysis. CID Ar partial pressure in the Q2 cell was set to 270 kPa. The electrode voltage of Q1 pre bias, collision cell Q2, and Q3 pre bias, and parent and fragment ion *m/z* were set using the optimization support software (LabSolutions, Shimadzu). For MRM transition, one fragment ion of b‐ or y‐series was selected for quantitation, and two ions were selected for structural confirmation according to the optimized MRM ion yield (Table 1).

**Table 1:** Optimal MRM transition of Etanercept and Abatacept signature peptides for bioanalytical validation

Candidate signature peptides of Etanercept						
Selected peptide	aa	Optimal MRM condition				Role
		Transition mass filter [*m/z*]	Q1 [V]	Collision [V]	Q3 [V]	
VFCTK	42‐46	299.3→498.2 (y4^+^) 299.3→351.2 (y3^+^) 299.3→247.9 (y2^+^)	−30	−12 −12 −18	−19 −25 −12	Quantitation Structure Structure
LPAQVAF TPYAPEP GSTCR	1‐18	669.2→423.7 (y8^++^) 669.2→580.3 (b6^+^) 669.2→846.4 (y8^+^)	−40	−16 −14 −19	−23 −22 −32	Quantitation Structure Structure

Candidate signature peptide of Abatacept						
Selected peptide	aa	Optimal MRM condition				Role
		Transition mass filter [*m/z*]	Q1 [V]	Collision [V]	Q3 [V]	
MHVAQPA VVLASSR	1‐13	489.3→420.2 (y4^+^) 489.3→834.4 (b8^+^) 489.3→567.3 (b5^+^)	−30	−16 −17 −21	−30 −30 −20	Quantitation Structure Structure

The parameters are defined as follows: Selected peptide; peptide sequence for quantitation, aa; amino acid position of selected peptide, Transition mass filter; fragment ion *m/z* for quantitation from the parent ion *m/z*, Q1 [V]; voltage condition of the quadrupole cell Q1, Collision; electrode voltage of collision cell Q2, Q3 [V]; voltage condition of the quadrupole cell Q3, Role; purpose of each ion *m/z*.

### 2.5 Oxidation tolerance of cysteine in Etanercept signature peptide VFCTK

The synthetic peptide VFCTK (1 mmol/L in 0.1% formic acid) was mixed with 1 mmol/L hydrogen peroxide (H_2_O_2_) in 25 mmol/L Tris‐HCl buffer pH 8.0. This reaction mixture was incubated for 30 minutes at room temperature for the cysteine oxidation reaction. After oxidation, the reaction mixture was diluted at 10‐fold in nSMOL reaction solution of 25 mmol/L Tris buffer pH 8.5 with 2M urea and 0.2 mmol/L TCEP, or 25 mmol/L Tris buffer pH 8.0. Peptide VFCTK stability verification was carried out for 5 hours at 50°C with gentle vortexing. The stability test reaction was quenched by adding formic acid at a final concentration of 0.5%. The monomer VFCTK with free thiol and the oxidized dimer with disulfide bridge was quantified by MRM analysis using individual optimized transition of a precursor ion *m/z* 299.15 [M + 2H]2 +  and product *m/z* 498.35 [y4]+, and of a precursor *m/z* 398.10 [M + 3H]3 +  and product *m/z* 497.30 [y4]+ after disulfide cleavage, respectively. Each MRM quantitation was normalized by adding 10 fmol of P14R ISTD intensity (Table 2).

**Table 2:** The results of oxidation tolerance of cysteine in the synthetic Etanercept signature peptide VFCTK

Peptide condition	Ratio of monomer (MRM transition of *m/z* 299.15 → 498.35)	Ratio of oxidized dimer (MRM transition of *m/z* 398.10 → 497.30)
Control	100	N.A.*
1 mmol/L H_2_O_2_ treatment	0.255	100
In nSMOL reaction solution	95.6	N.D.*
In Tris buffer, pH 8.0	81.6	12.3
nSMOL reaction solution after H_2_O_2_ treatment	97.7	N.D.
Tris buffer after H_2_O_2_ treatment	0.183	82.1

*N.A.: not analyzed, N.D.: not detected.

### 2.6 The content determination of N‐terminal peptide heterogeneity in Abatacept

In order to determine the ratio of the N‐terminal peptide content of Abatacept, MRM optimization was performed using three synthetic candidate peptides MHVAQPAVVLASSR, AMHVAQPAVVLASSR, and MAMHVAQPAVVLASSR (summarized in Table 3). Information about each Abatacept peptide was obtained from DrugBank, Review report from Pharmaceuticals and Medical Devices Agency (PMDA) Japan, and predicted from KEGG Drug, respectively. And then, nSMOL quantitation of Abatacept (10 and 100 μg/mL) was carried out in PBS buffer and in human serum. Each MRM intensity was normalized by 10 fmol of P14R ISTD intensity (Table 4).

**Table 3:** The potential heterogeneity of the Abatacept N‐terminal peptide

Peptide sequence	Optimal MRM condition				Registered from
	Transition mass filter [*m/z*]	Q1 [V]	Collision [V]	Q3 [V]	
MHVAQPAVVLASSR	489.3→420.2 (y4^+^)	−30	−16	−30	DrugBank
AMHVAQPAVVLASSR	513.0→420.2 (y4^+^)	−30	−18	−30	Review report
MAMHVAQPAVVLASSR	556.0→420.2 (y4^+^)	−30	−17	−30	Estimated from KEGG Drug

**Table 4:** The content of three potential N‐terminal peptides on Abatacept against peptide MHVAQPAVVLASSR

Peptide candidate	In PBS	In human serum
MHVAQPAVVLASSR	100	100
AMHVAQPAVVLASSR	37.0	38.1
MAMHVAQPAVVLASSR	4.87	5.42

### 2.7 Preparation of sample for validation by nSMOL proteolysis

In the current study, we performed a bioanalytical validation of Etanercept or Abatacept in human serum using the nSMOL method as described in our previous report with a minor improvement. The nSMOL proteolysis coupled with the LC‐MS/MS method was validated in accordance with the Guideline on Bioanalytical Method Validation in Pharmaceutical Development from Notification 0711‐1 of the Evaluation and Licensing Division, Pharmaceutical and Food Safety Bureau, the Ministry of Health, Labour and Welfare, dated July 11, 2013. The objective of a full validation is to demonstrate the assay performance of the method, eg, selectivity, lower limit of quantification (LLOQ), calibration curve, accuracy, precision, matrix effect, carryover, dilution integrity, and stored and processed sample stability. Briefly, all validation sample sets were prepared and stored at −20°C or −80°C for 24 hours or longer before each validation assay. A 20 μL aliquot of the Fc‐fusion protein‐spiked human serum was diluted 10‐fold in PBS (pH 7.4) containing 0.1% OTG for avoiding a nonspecific binding to the resin and plastic materials. The IgG fraction from serum sample was collected with 50 μL of PBS‐substituted AF‐rProtein A resin (50% slurry) in 180 μL of PBS containing OTG with gentle vortexing at 25°C for 15 minutes. Protein A resin was harvested onto Ultrafree filter and washed twice with 300 μL of PBS containing OTG for removing other serum proteins except for IgGs, and then with 300 μL of PBS for removing detergents that inhibit column separation, carryover, and ionization of peptides in ESI interface. Each washing substitution was directly performed by centrifugation (10,000*g* for 1 minutes) on filter devices. After washing step, Protein A resin was substituted with 150 μL of 25 mmol/L Tris‐HCl (pH8.5) containing 10 fmol/μL P14R, 2 mol/L urea, and 0.2 mmol/L TCEP for keeping mild reducing condition and preventing oxidative binding of free thiol. nSMOL proteolysis was carried out using 20 μg trypsin on FG‐beads with gentle vortexing at 50°C for 5 hours in saturated vapor atmosphere for uniform contact between Protein A resin and FG‐beads nanoparticles. After nSMOL proteolysis, the reaction was stopped by adding formic acid at a final concentration of 0.5%. The peptide solution was collected by centrifugation (10,000*g* for 1 minutes) to remove Protein A resin and trypsin FG‐beads. These analytes were transferred into low protein‐binding polypropylene vials, and then performed by LC‐MS analysis. The concentration of Etanercept and Abatacept in serum samples was set from 0.195 to 100 μg/mL, and from 0.391 to 100 μg/mL with 2‐fold serially dilution for 10 calibration samples, respectively. The concentrations of LLOQ, low quality control (LQC), middle quality control (MQC), and high quality control (HQC) for Etanercept were 0.195, 0.586, 10.0, and 100 μg/mL. And the concentration set for Abatacept were 0.391, 0.586, 10.0, and 100 μg/mL, respectively (Table 5, 6, and Supplementary Information).

**Table 5:** The summary of the precision and accuracy of Etanercept VFCTK in inter‐ and intraday assay

Assay	Nominal concentration	Set concentration (μg/mL)			
		0.195	0.586	9.38	80.0
Run 1 (N = 5)	Mean	0.202	0.590	9.90	84.9
	SD	0.02	0.05	0.41	4.52
	CV (%)	11.2	7.89	4.17	5.32
	Accuracy (%)	104	101	106	106
Run 2 (N = 5)	Mean	0.192	0.583	9.26	78.7
	SD	0.01	0.03	0.22	2.86
	CV (%)	7.57	5.33	2.37	3.64
	Accuracy (%)	98.6	100	98.7	98.3
Run 3 (N = 5)	Mean	0.196	0.571	10.2	87.0
	SD	0.01	0.02	0.29	2.69
	CV (%)	7.29	3.80	2.83	3.09
	Accuracy (%)	100	97.4	109	109
Average (N = 15)	Mean	0.197	0.581	9.79	83.5
	SD	0.02	0.03	0.50	4.9
	CV (%)	8.57	5.69	5.13	5.84
	Accuracy (%)	101	99.2	104	104

**Table 6:** The summary of precision and accuracy of Abatacept MHVAQPAVVLASSR in inter‐ and intraday assay

Assay	Nominal concentration	Set concentration (μg/mL)			
		0.391	0.586	9.38	80.0
Run 1 (N = 5)	Mean	0.422	0.539	8.42	68.4
	SD	0.0321	0.0390	0.482	2.51
	CV (%)	7.6	7.24	5.72	3.67
	Accuracy (%)	108	92.0	89.8	85.5
Run 2 (N = 5)	Mean	0.340	0.540	8.56	74.5
	SD	0.0506	0.0624	0.387	2.69
	CV (%)	14.9	11.6	4.52	3.60
	Accuracy (%)	86.9	92.2	91.3	93.2
Run 3 (N = 5)	Mean	0.397	0.484	8.69	71.2
	SD	0.0427	0.0290	0.660	2.17
	CV (%)	10.8	5.99	7.59	3.04
	Accuracy (%)	101	82.5	92.7	89.0
Average (N = 15)	Mean	0.386	0.521	8.56	71.4
	SD	0.00929	0.0172	0.139	0.264
	CV (%)	2.41	3.29	1.62	0.370
	Accuracy (%)	98.7	88.9	91.3	89.2

## 3 RESULTS AND DISCUSSION

### 3.1 nSMOL reaction yield of Fc‐fusion protein pharmaceuticals

Structural properties of monoclonal antibodies and Fc‐fusion protein Etanercept are quite different in terms of molecular dynamics, occupied diameter, and fluctuation on hinge function. The main shape of antibodies consists of two distinctive heavy‐ and light‐chains with inter‐ and intradisulfide bonding. Antibodies have a variable domain (Fv) in each N‐terminus region, and following constant framework structure (CH1, CH2, and CH3 in the heavy chain, and CL in the light chain). These features are not markedly different depending on the immunoglobulin G molecular family. On the other hand, Ethanercept and Abatacept consist of extracellular domains of TNFR and CTLA‐4, respectively. These molecules have a highly complexed structure by disulfide formation. Moreover, these receptors express their physiological functions by dimerization or trimerization.^28^, ^29^ Additionally, these Fc‐fusion proteins have no hinge‐like portions between the Fc and fused domains. Therefore, we analyzed the reaction yield of Etanercept using the nSMOL method. A densitometric analysis on SDS‐PAGE showed that the recovery rate of Etanercept by Protein A resin was calculated at 99.1%, and the retention rate on Protein A after nSMOL reaction condition was 93.1%, as given in the Supplementary Information. After nSMOL proteolysis, full length of Etanercept was not detected. This result indicated that a domain‐selective nSMOL reaction on the N‐terminus of Fc‐fusion proteins was successfully proceeded independent of the difference of structural chemistry and molecular dynamics from IgG family.

### 3.2 The limitation regarding the selection of signature peptide for Fc‐fusion biopharmaceuticals

**Figure 2:**
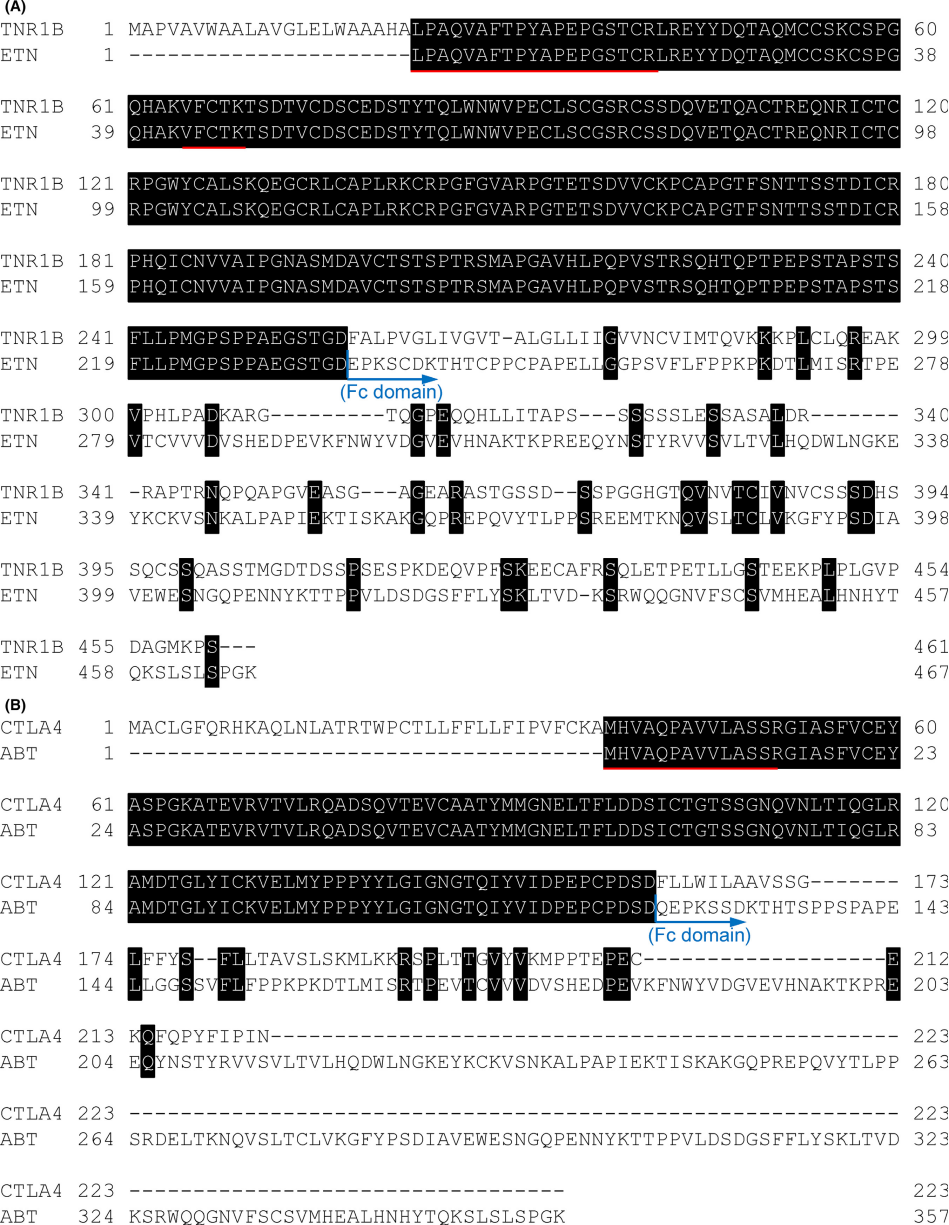
The ClustalW sequence alignment of (A) TNFR (TNR1B) and Etanercept (ETN), and (B) CTLA‐4 (CTLA4) and Abatacept (ABT). The black area shows identical amino acid residues. The red lines show the selected signature peptide of each Fc‐fusion protein. The blue arrow represents the position of the beginning of fused Fc domain

**Figure 3:**
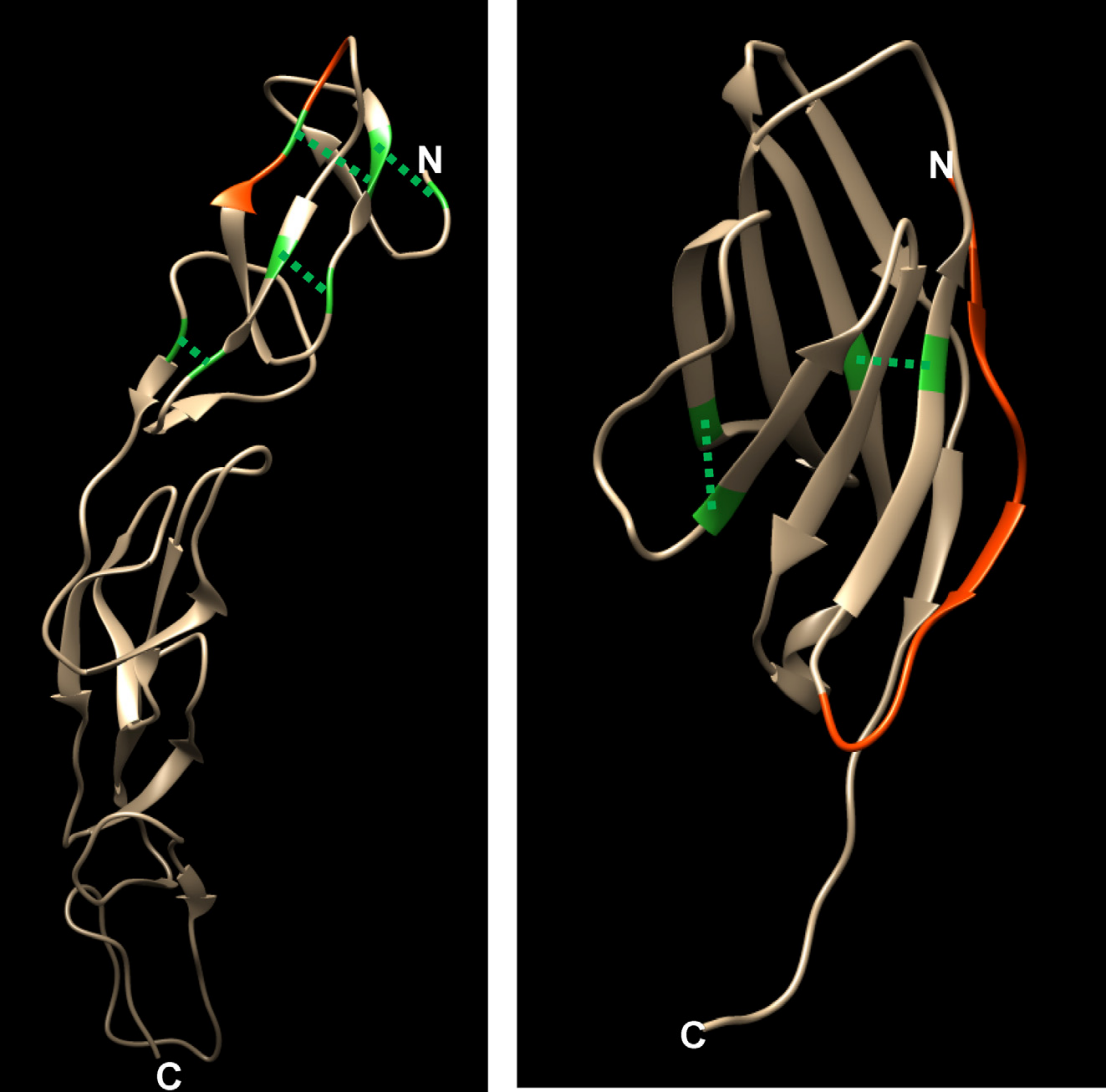
The 3‐D structure of the extracellular domain and signature peptide configuration of (A) TNFR (from Protein Data Bank ID 3ALQ), peptide VFCTK (aa.42‐46), and (B) CTLA‐4 (PDB ID 3OSK), peptide MHVAQPAVVLASSR (aa.1‐13). The red position shows the selected signature peptide. Green residues indicate cysteine, and the dashed lines show the site of intradisulfide bridge

We have previously defined that the signature peptide of antibody drugs should be selected as follows: peptides with from 8 to 15 amino acid residues, with no cysteine residue, with no missed cleavage in the tryptic reaction, not in the vicinity of the disulfide bonding, with specific sequences containing CDR sequences against endogenous IgGs, and with no N‐ and C‐terminus sequences because of amino acid heterogeneity on terminal fragment.^30^ However, for performing the Etanercept and Abatacept assay development using LC‐MS, we could not select the signature peptide according to our criteria because the selection of signature peptide was markedly limited by the complexed disulfide structure and low content of lysine and arginine residues aligned by ClustalW analysis shown in Figure 2. As a consequence, there was no choice but to select the cysteine‐containing peptide of Etanercept and N‐terminus peptide of Abatacept as candidate signature peptides for the nSMOL bioanalysis, respectively (Figure 3).

### 3.3 The stability of cysteine‐containing peptide as a signature peptide of Etanercept in LC‐MS bioanalysis

The representative oxidative product of the cysteine‐containing peptide is formed by the dimerization via disulfide bridge formation. Therefore, we verified the ratio of the reduced and oxidative form, reversibility, stability, and oxidative tolerance in nSMOL reaction using a H_2_O_2_‐treated dimerization peptide VFCTK. These results are summarized in Table 2. The oxidative dimerization of VFCTK by H_2_O_2_ in Tris buffer was performed, but this dimerization rearrangement was not observed in the nSMOL reaction solution. More than 10% of peptides underwent the oxidative dimerization in normal Tris buffer conditions, suggesting that this could affect the quantitative data in nSMOL bioanalysis using a normal buffer condition. On the other hand, the dimerized peptide did not dissociate to the monomer form by reductive cleavage in Tris buffer circumstances, whereas the reductive monomerization was occurred successfully in the nSMOL reaction solution, indicating that this nSMOL condition made stable quantitation in the LC‐MS assay possible. We considered that a stable bioanalysis using the nSMOL principle would even be possible using a cysteine‐containing peptide.

### 3.4 The heterogeneity of the N‐terminus containing peptide

**Figure 4:**
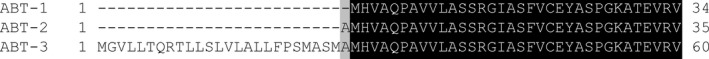
The ClustalW alignment of reported N‐terminal Abatacept sequences. ClustalW alignment of the N‐terminal portion from DrugBank (ABT‐1), Review report from PMDA (ABT‐2), and KEGG drug (ABT‐3) is shown

There are several reports on the N‐terminal sequence of Abatacept.^31^ We investigated the sequence information from two databases (DrugBank and KEGG Drug) and one document (PMDA) in Figure 4. Therefore, we have determined the ratio of the N‐terminal peptide sequences. Since Abatacept is produced from genetically engineered CHO cells, we have assumed that the three potential peptide sequences, MHVAQPAVVLASSR, AMHVAQPAVVLASSR, and MAMHVAQPAVVLASSR, were assigned. For the quantitation of these peptides, we have determined the sequence ratio by monitoring the common fragment signature y4 ion series because the fragmentation energies on the y4 position in the collision cell Q2 were expected to be the same. Additionally, it is possible to compare the quantitative data with peptide structural observations even if the ionization energy of the individual precursor is different. The quantitative analysis showed in Table 4 indicates that the most frequent peptide was MHVAQPAVVLASSR at about a 60% content, and this ratio was constant and not degraded during the nSMOL reaction condition in PBS buffer and serum. Therefore, we have decided to use the peptide MHVAQPAVVLASSR for further validation assay.

### 3.5 Summary of the validation assay of Etanercept and Abatacept by the nSMOL approach

A complete validation LC‐MS bioanalysis for Etanercept and Abatacept was performed by the nSMOL, in accordance with the Guideline on Bioanalytical Method Validation in Pharmaceutical Development. In Tables 5 and 6, data for precision and accuracy are briefly summarized. And all the validated data are shown in the Supplementary Information section. And representative MRM spectra of blank, zero, and LLOQ sample for Etanercept and Abatacept are shown in the Supplementary Information, respectively. All the validated dataset met the guideline criteria from the concentration range of 0.195 to 100 μg/mL for Etanercept, and from 0.391 to 100 μg/mL for Abatacept in human serum, indicating that the LC‐MS bioanalysis of Etanercept and Abatacept may be satisfactory, and could be applied to therapeutic drug monitoring and clinical pharmacokinetic studies.

In conclusion, we have developed a new validated LC‐MS bioanalysis for the Fc‐fusion biopharmaceuticals Etanercept and Abatacept, using the nSMOL application like the case for several antibody drugs reported previously.^32^, ^33^, ^34^ To our knowledge, this is the first study to apply a direct quantitation of Etanercept and Abatacept in human serum using validated LC‐MS bioanalysis. The cysteine‐containing peptides from the Fc‐fusion proteins can be analyzed with sufficient tolerance to oxidative modifications in the nSMOL bioanalysis procedures, and the N‐terminal peptides with amino acid heterogeneity can also be analyzed using the content of the most abundant structures. The issue of protein bioanalysis using LC‐MS is summarized in how to keep the robustness of the instruments. And for reproducible practice in clinical studies, the issue should be solved by overall optimization from sample prep to chromatograph, and mass spectrometry. The essential advantages of nSMOL approach, in principle, are keeping structural specificity of substrates while decreasing a large excess of peptide analytes. We indicate that nSMOL chemistry might have one of the potential methodologies to solve these LC‐MS applications in clinical field. Our present report demonstrates that Fab‐selective reaction nSMOL proteolysis would be expected as a global and powerful tool for the regulatory LC‐MS bioanalysis of monoclonal antibodies and Fc‐fusion biopharmaceuticals for various pharmacokinetic, clinical, and therapeutic scene.

## DISCLOSURES

NI, KY, MT, and TS are employees of SHIMADZU Corporation. And the authors have no conflicts of interest directly relevant to the content of this article.
