# Supplementary material for: Application of nSMOL coupled with LC‐MS bioanalysis for monitoring the Fc‐fusion biopharmaceuticals Etanercept and Abatacept in human serum
Source: Pharmacol Res Perspect. 2018 Jul 24;6(4):e00422. doi: 10.1002/prp2.422 (PMC6056752; doi:10.1002/prp2.422)
Supplement: Supplementary file 1 [file PRP2-6-e00422-s001.pdf]

Supplemental online material

Application of nSMOL coupled with LC-MS bioanalysis for monitoring the Fc-fusion biopharmaceuticals Etanercept and Abatacept in human serum

Noriko Iwamoto[1], Kotoko Yokoyama[1], Megumi Takanashi[1], Atsushi Yonezawa[2,3], Kazuo Matsubara[2], and Takashi Shimada[1]*

1. Leading Technology of Bioanalysis and Protein Chemistry, SHIMADZU Corporation
2. Department of Clinical Pharmacology and Therapeutics, Kyoto University Hospital
3. Graduate School of Pharmaceutical Sciences, Kyoto University

Correspondence to:

Takashi Shimada, Ph.D.
SHIMADZU Corporation
46-29 Yoshida-Shimo-Adachi-cho, Sakyo-ku, Kyoto 606-8501, Japan
Med-Pharm Collaboration Building of Kyoto University
Tel: +81-75-708-6820
Fax: +81-75-708-8084
Email: t-shima@shimadzu.co.jp

Supplementary table

These tables are described the result of the fully validated LC-MS bioanalysis of Ethanercept and Abatacept using nSMOL proteolysis.

Table S1. Calibration curve of Etanercept VFCTK

Nominal concentration (μg/ml)	Back-calculated concentration (μg/ml)			Accuracy (%)		
	1	2	3	1	2	3
0.195	0.193	0.202	0.198	98.9	104	101
0.391	0.404	0.377	0.389	103	96.4	99.4
0.781	0.798	0.727	0.797	102	93.1	102
1.56	1.57	1.45	1.41	101	93.0	90.4
3.13	3.07	3.36	2.93	98.0	107	93.5
6.25	6.10	6.16	6.02	97.6	98.6	96.2
12.5	12.7	12.9	12.8	102	103	103
25.0	23.8	25.3	26.9	95.0	101	108
50.0	52.5	50.3	55.5	105	101	111
100	99.2	108	103	99.2	108	103

Table S2. Precision and accuracy of Etanercept VFCTK

Run	Nominal concentration	Concentration (μg/ml)			
		0.195	0.586	9.38	80.0
1	Observed	0.199	0.629	10.1	87.0
		0.233	0.612	10.4	88.7
		0.194	0.606	9.33	88.8
		0.213	0.592	9.68	80.3
		0.172	0.510	9.98	79.8
	Mean	0.202	0.590	9.90	84.9
	SD	0.02	0.05	0.41	4.52
	CV (%)	11.2	7.89	4.17	5.32
	Accuracy (%)	104	101	106	106
2	Observed	0.185	0.581	8.89	81.6
		0.215	0.570	9.29	74.9
		0.176	0.540	9.48	77.9
		0.190	0.618	9.26	81.5
		0.195	0.608	9.36	77.4
	Mean	0.192	0.583	9.26	78.7
	SD	0.01	0.03	0.22	2.86
	CV (%)	7.57	5.33	2.37	3.64
	Accuracy (%)	98.6	100	98.7	98.3
3	Observed	0.205	0.555	9.85	87.7
		0.183	0.572	9.93	89.1
		0.186	0.553	10.32	84.4
		0.188	0.568	10.40	84.1
		0.216	0.607	10.49	89.9
	Mean	0.196	0.571	10.2	87.0
	SD	0.01	0.02	0.29	2.69
	CV (%)	7.29	3.80	2.83	3.09
	Accuracy (%)	100	97.4	109	109
	Mean (N=15)	0.197	0.581	9.79	83.5
	SD (N=15)	0.02	0.03	0.50	4.9
	CV (%)	8.57	5.69	5.13	5.84
	Accuracy (%)	101	99.2	104	104

Table S3. Matrix effect of Etanercept VFCTK

Analyte	Corresponding concentration (μg/ml)	Blank matrix No.	$P_{14}R$-normalized MF	Mean	SD	CV (%)
Etanercept	0.586	M1	6.62	6.83	0.454	6.65
		M2	7.72			
		M3	6.89			
		F1	6.51			
		F2	6.57			
		F3	6.66			
	80.0	M1	1.32	1.32	0.0293	2.22
		M2	1.31			
		M3	1.34			
		F1	1.27			
		F2	1.32			
		F3	1.35			

Table S4. Carryover of Etanercept VFCTK

Compound	Run	Peak area		Peak area rate (%)
		LLOQ	Carry over sample	
Etanercept	1	1571	0	0.0
	2	1622	0	0.0
	3	1337	0	0.0
$P_{14}R$	1	308139	0	0.0

Table S5. Dilution integrity of Etanercept VFCTK

Nominal concentration (μg/ml)	Dilution factor	Observed* (μg/ml)	Mean	SD	CV (%)	Accuracy (%)
500	10	47.1	474	0.65	1.37	94.9
		47.1				
		48.0				
		48.2				
		46.8				
500	25	18.9	475	0.88	4.63	95.1
		20.5				
		18.2				
		19.0				
		18.5				

Table S6. Confirmation of QC sample for stability of Etanercept VFCTK

Parameters for stability studies	Concentrations of Etanercept in human plasma (μg/ml)			
	0.586		80.0	
	Mean (μg/ml)	Accuracy (%)	Mean (μg/ml)	Accuracy (%)
Stability in plasma during freeze (-30°C) and thaw cycles				
Cycle 5	0.541	92.4	81.6	102
Stability in plasma during freeze (-80°C) and thaw cycles				
Cycle 5	0.528	90.2	83.2	104
Short-term stability in plasma for 4 hours at room temperature				
	0.536	91.4	78.4	98.0
Long-term stability in plasma for 30 days at -30°C				
	0.546	93.2	81.7	102
Long-term stability in plasma for 30 days at -80°C				
	0.575	98.1	79.7	100
Processed sample stability in HPLC set at 5°C				
For 24 hours	0.594	101	75.1	93.8
For 48 hours	0.570	97.3	80.9	101

Table S7. Calibration curve of Etanercept LPAQVAFTPYAPEPGSTCR

Nominal concentration (μg/ml)	Back-calculated concentration (μg/ml)			Accuracy (%)		
	1	2	3	1	2	3
0.195	0.194	0.187	0.214	99.3	95.9	110
0.391	0.392	0.430	0.338	100	110	86.4
0.781	0.814	0.899	0.794	104	115	102
1.56	1.57	1.60	1.56	101	103	100
3.13	3.07	3.10	3.23	98.1	98.9	103
6.25	6.52	6.04	6.13	104	96.6	98.1
12.5	12.6	12.2	13.2	101	98.0	105
25	23.3	23.2	24.3	93.3	93.0	97.3
50	51.7	50.9	51.3	103	102	103
100	97.7	96.2	103	97.7	96.2	103

Table S8. Precision and accuracy of Etanercept LPAQVAFTPYAPEPGSTCR

Run	Nominal concentration	Concentration (μg/ml)			
		0.195	0.586	9.38	80.0
1	Observed	0.222	0.560	9.80	88.6
		0.191	0.543	10.17	89.0
		0.165	0.551	9.59	86.1
		0.178	0.529	9.65	85.3
		0.159	0.611	9.65	78.5
	Mean	0.183	0.559	9.77	85.5
	SD	0.03	0.03	0.24	4.19
	CV (%)	13.69	5.61	2.41	4.90
	Accuracy (%)	93.8	95.4	104	107
2	Observed	0.166	0.604	9.69	77.4
		0.203	0.583	9.54	77.6
		0.171	0.629	9.54	76.4
		0.194	0.622	9.42	79.9
		0.174	0.611	9.64	80.2
	Mean	0.182	0.610	9.57	78.3
	SD	0.02	0.02	0.10	1.65
	CV (%)	8.82	2.92	1.08	2.11
	Accuracy (%)	93.1	104	102	97.9
3	Observed	0.187	0.587	9.43	81.1
		0.191	0.512	9.46	80.6
		0.191	0.504	9.95	77.7
		0.181	0.566	9.37	78.3
		0.179	0.507	9.92	82.9
	Mean	0.186	0.535	9.63	80.1
	SD	0.01	0.04	0.28	2.15
	CV (%)	3.01	7.20	2.94	2.68
	Accuracy (%)	95.3	91.3	103	100
	Mean (N=15)	0.18	0.57	9.65	81.3
	SD (N=15)	0.02	0.04	0.22	4.14
	CV (%)	8.87	7.54	2.32	5.09
	Accuracy (%)	94.1	96.9	103	102

Table S9. Matrix effect of Etanercept LPAQVAFTPYAPEPGSTCR

Analyte	Corresponding concentration (μg/ml)	Blank matrix No.	$P_{14}R$-normalized MF	Mean	SD	CV (%)
Etanercept	0.586	M1	4.95	4.80	0.151	3.15
		M2	4.82			
		M3	4.89			
		F1	4.51			
		F2	4.83			
		F3	4.79			
	80.0	M1	1.14	1.15	0.0474	4.13
		M2	1.14			
		M3	1.24			
		F1	1.12			
		F2	1.14			
		F3	1.11			

Table S10 Carryover of Etanercept LPAQVAFTPYAPEPGSTCR

Compound	Run	Peak area		Peak area rate (%)
		LLOQ	Carry over sample	
Etanercept	1	2324	267	11.5
	2	2310	425	18.4
	3	2385	179	7.51

ND: Not detected

NC: Not calculated

Table S11. Confirmation of QC sample for stability of Etanercept LPAQVAFTPYAPEPGSTCR

Parameters for stability studies	Concentrations of Etanercept in human plasma (μg/ml)			
	0.586		80.0	
	Mean (μg/ml)	Accuracy (%)	Mean (μg/ml)	Accuracy (%)
Stability in plasma during freeze (-30°C) and thaw cycles				
Cycle 5	0.632	108	79.5	99.4
Stability in plasma during freeze (-80°C) and thaw cycles				
Cycle 5	0.567	96.8	81.6	102
Short-term stability in plasma for 4 hours at room temperature				
	0.612	104	78.8	98.5
Long-term stability in plasma for 30 days at -30°C				
	0.544	92.8	78.9	98.6
Long-term stability in plasma for 30 days at -80°C				
	0.559	95.4	79.7	99.7
Processed sample stability in HPLC set at 5°C				
For 24 hours	0.585	99.8	77.9	97.4
For 48 hours	0.564	96.2	77.8	97.3

Table S12. Calibration curve of Abatacept MHVAQPAVVLASSR

Nominal concentration (μg/ml)	Back-calculated concentration (μg/ml)			Accuracy (%)		
	1	2	3	1	2	3
0.391	0.386	0.370	0.407	98.7	94.7	104
0.781	0.827	0.886	0.763	106	113	97.7
1.56	1.49	1.74	1.43	95.6	112	91.9
3.13	3.06	3.09	3.18	97.7	98.8	102
6.25	6.46	6.11	6.26	103	97.7	100
12.5	12.5	13.0	12.6	100	104	101
25.0	25.5	22.5	24.6	102	90.1	98.4
50.0	54.1	49.1	50.7	108	98.2	101
100	92.6	100	107	92.6	101	107

Table S13. Precision and accuracy of Abatacept MHVAQPAVVLASSR

Run	Nominal concentration	Concentration (μg/ml)			
		0.391	0.586	9.38	80.0
1	Observed	0.470	0.507	8.03	65.4
		0.432	0.604	8.45	69.0
		0.387	0.537	9.24	68.7
		0.419	0.538	8.17	66.8
		0.400	0.510	8.22	72.0
	Mean	0.422	0.539	8.42	68.4
	SD	0.0321	0.0390	0.482	2.51
	CV (%)	7.61	7.24	5.72	3.67
	Accuracy (%)	108	92.0	89.8	85.5
2	Observed	0.370	0.559	9.07	75.7
		0.291	0.507	8.69	73.8
		0.403	0.518	8.17	75.3
		0.348	0.639	8.17	77.6
		0.286	0.478	8.69	70.4
	Mean	0.340	0.540	8.56	74.5
	SD	0.0506	0.0624	0.387	2.69
	CV (%)	14.9	11.6	4.52	3.60
	Accuracy (%)	86.9	92.2	91.3	93.2
3	Observed	0.338	0.506	9.71	70.1
		0.423	0.512	8.89	68.5
		0.400	0.493	7.96	74.1
		0.448	0.445	8.56	71.0
		0.374	0.462	8.35	72.5
	Mean	0.397	0.484	8.69	71.2
	SD	0.0427	0.0290	0.660	2.17
	CV (%)	10.8	5.99	7.59	3.04
	Accuracy (%)	101	82.5	92.7	89.0
	Mean (N=15)	0.386	0.521	8.56	71.4
	SD (N=15)	0.00929	0.0172	0.139	0.264
	CV (%)	2.41	3.29	1.62	0.370
	Accuracy (%)	98.7	88.9	91.3	89.2

Table S14. Matrix effect of Abatacept MHVAQPAVVLASSR

Analyte	Corresponding concentration (μg/ml)	Blank matrix No.	$P_{14}R$-normalized MF	Mean	SD	CV (%)
Etanercept	0.586	M1	7.08	7.09	0.438	6.18
		M2	6.96			
		M3	7.29			
		F1	7.82			
		F2	6.85			
		F3	6.53			
	80.0	M1	1.48	1.47	0.0504	3.42
		M2	1.45			
		M3	1.56			
		F1	1.48			
		F2	1.46			
		F3	1.41			

Table S15. Carryover of Abatacept MHVAQPAVVLASSR

Compound	Run	Peak area		Peak area rate (%)
		LLOQ	Carry over sample	
Etanercept	1	9722	1712	17.6
	2	10651	1582	14.9
	3	10391	1416	13.6
$P_{14}R$	1	190449	0	0

Table S16. Dilution integrity of Abatacept MHVAQPAVVLASSR

Nominal concentration (µg/ml)	Dilution factor	Observed* (µg/ml)	Mean	SD	CV (%)	Accuracy (%)
500	10	48.8	455	2.33	5.12	90.9
		46.2				
		43.4				
		43.0				
		46.0				
500	25	18.4	473	0.832	4.39	94.7
		19.2				
		20.3				
		18.3				
		18.5				

Table S17. Confirmation of QC sample for stability of Abatacept MHVAQPAVVLASSR

Parameters for stability studies	Concentrations of Etanercept in human plasma (µg/ml)			
	0.586		80.0	
	Mean (µg/ml)	Accuracy (%)	Mean (µg/ml)	Accuracy (%)
Stability in plasma during freeze (-30°C) and thaw cycles				
Cycle 5	0.595	102	79.5	99.4
Stability in plasma during freeze (-80°C) and thaw cycles				
Cycle 5	0.537	91.6	82.4	103
Short-term stability in plasma for 4 hours at room temperature				
	0.599	102	71.8	89.7
Long-term stability in plasma for 30 days at -30°C				
	0.633	108	81.1	101
Long-term stability in plasma for 30 days at -80°C				
	0.623	106	79.3	99.1
Processed sample stability in HPLC set at 5°C				
For 24 hours	0.570	97.3	77.2	96.5
For 48 hours	0.590	101	77.6	97.1

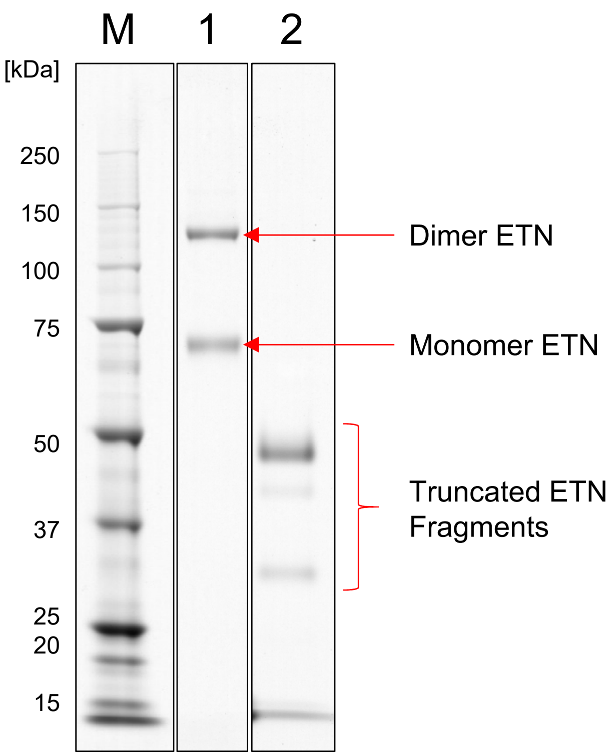

	1	2
Lane Density	830	1016
%	100	122

M: Molecular weight marker
1: ETN control
2: ETN on Protein A resin after nSMOL reaction with FG trypsin beads

Figure S1. The densitometric analysis of Etanercept reaction yield in nSMOL procedure. SDS-PAGE separated bands were visualized by CBB-G250 staining solution. The gel image was acquired by GS-900 Calibrated Densitometer and analyzed by ImageLab software (BioRad). The calculated total lane density are described in the table.

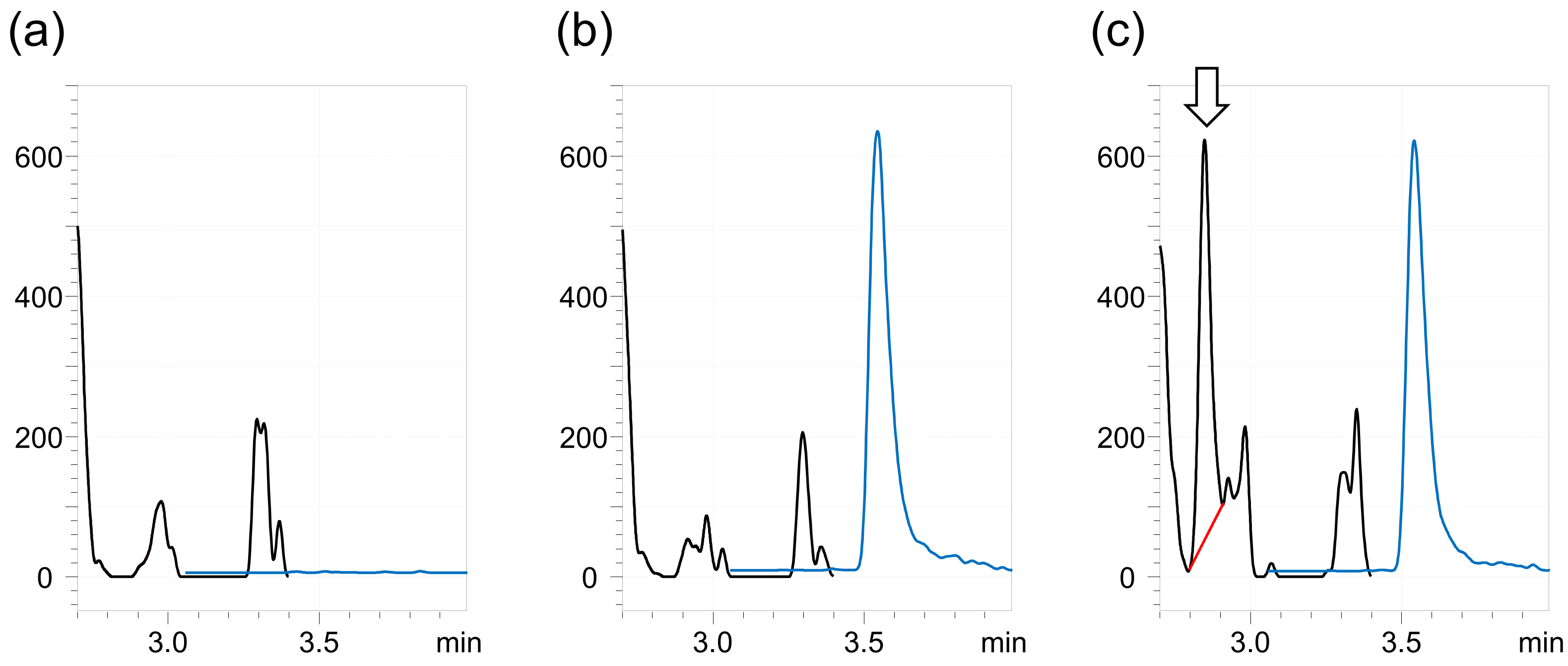

Figure S2. Representative MRM chromatogram VFCTK from Etanercept of (a) blank serum sample, (b) blank human serum spiked with the internal standards, and (c) blank human serum spiked with Etanercept at LLOQ. The black line shows MRM chromatogram of VFCTK and arrow is ETN-derived peak. The blue line is P14R internal standard with relative intensity.

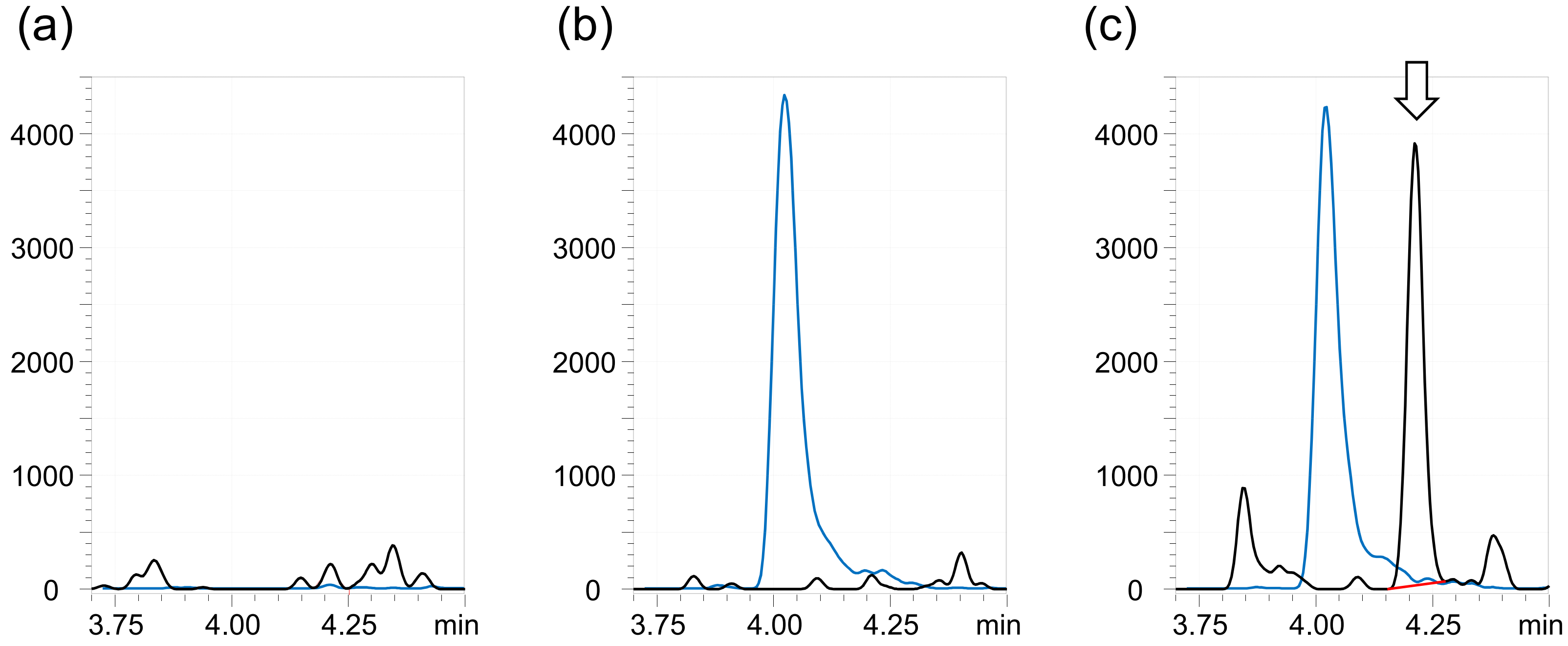

Figure S3. Representative MRM chromatogram MHVAQPAVVLASSR from Abatacept of (a) blank serum sample, (b) blank human serum spiked with the internal standards, and (c) blank human serum spiked with Abatacept at LLOQ. The black line shows MRM chromatogram of MHVAQPAVVLASSR and arrow is ABT-derived peak. The blue line is P14R internal standard with relative intensity.
